# Evaluating the Necessity of Routine Histopathology in Sleeve Gastrectomy Specimens: A Five-Year Analysis

**DOI:** 10.7759/cureus.69666

**Published:** 2024-09-18

**Authors:** Muhammad Ali Khattak, Ahmed Muhammad Rafique, Yasir Iqbal, Habeeb Abdulrasheed, Muhammad Usman Khan, Amman Malik

**Affiliations:** 1 Urology, University Hospitals Birmingham NHS Foundation Trust, Birmingham, GBR; 2 Surgery, University Hospitals Birmingham NHS Foundation Trust, Birmingham, GBR; 3 Acute and General Medicine, Queen Elizabeth Hospital Birmingham, Birmingham, GBR; 4 Urology, Northampton General Hospital, Northampton, GBR

**Keywords:** body mass index: bmi, laparoscopic sleeve gastrectomy (lsg), oesophagogastroduodenoscopy (ogd), roux-en-y gastric bypass (rygb), upper gastrointestinal endoscopy (ogd)

## Abstract

Introduction: Laparoscopic sleeve gastrectomy (LSG) is a widely performed bariatric surgery that involves the removal of a portion of the stomach. Routinely, the resected gastric tissue is sent for histopathological examination to screen for malignancies or other significant pathological findings. However, the necessity of this routine practice remains uncertain. This study aims to evaluate the histopathological outcomes of LSG specimens over a five-year period at our institution.

Methods: We conducted a retrospective analysis of 203 patients who underwent LSG between January 2017 and December 2022 at Heartlands Hospital, University Hospitals Birmingham. Data collected included patient demographics, body mass index (BMI), use of preoperative oesophagogastroduodenoscopy (OGD), and histopathological findings. Patients with incomplete records or those who underwent Roux-en-Y gastric bypass were excluded from the study.

Results: Data were extracted for 310 patients, of whom 107 were excluded. The majority of the 203 patients analyzed were female (83%), with a mean age of 45.7 years and a mean BMI of 45.4 ± 7.3. Preoperative OGD was performed in only 0.5% of cases. Histopathological examination revealed that 81.3% (n=165) of patients had normal gastric mucosa, while 14.3% (n=29) had chronic gastritis. Clinically significant findings were rare, with only 1% (n=2) of patients showing gastrointestinal stromal tumors (GISTs) or focal intestinal metaplasia. None of the patients required additional treatment or follow-up based on these histopathological findings.

Conclusion: Most LSG specimens in our study showed normal or non-significant histopathological findings, raising questions about the routine use of histopathological examination in LSG procedures. It remains unclear whether histopathology is necessary following sleeve gastrectomy. While no patients in our cohort required further treatment or surveillance, there are reports in the literature where surveillance or further treatment was necessary, though the incidence remains low. Given the low incidence of clinically significant pathology, further studies with larger sample sizes and multi-center data are needed to establish clear guidelines on this issue.

## Introduction

Obesity has become a critical issue for public health and healthcare systems worldwide, due to its increasing occurrence not only in developed countries but also in lower-income regions. It imposes significant economic costs, linked to health-related issues like type II diabetes, cardiovascular diseases, cancer, and reduced life expectancy [1]. According to the World Health Organization (WHO), almost 39% of adults worldwide were overweight in 2016, nearly three times higher than in 1975. Of these, more than 650 million were classified as obese [2]. In cases of morbid obesity where diet and exercise are not effective, bariatric surgery offers a crucial treatment alternative. Laparoscopic sleeve gastrectomy (LSG) is among the most frequently conducted bariatric surgical procedures, appreciated for its comparatively low incidence of complications and mortality, as well as its favorable long-term weight loss outcomes [3].

Among various bariatric procedures, LSG is unique in providing a histopathology sample. Typically, the tissue samples obtained during these surgical procedures are found to be normal. However, in rare cases, pathological findings can lead to changes in management plans [4]. Most studies have reported that the majority of these LSG specimens had non-significant findings, adding an unnecessary burden for pathologists and increasing healthcare costs [5]. There are differing opinions regarding the management of histopathological specimens in bariatric surgery. One perspective recommends sending these remnants for histopathological analysis, while the other suggests it is unnecessary, as most findings are benign. Practices differ across various regions and centers [6].

Upper gastrointestinal endoscopy (oesophagogastroduodenoscopy, OGD) is a preferred diagnostic tool for individuals presenting with gastric symptoms. Nevertheless, it is not routinely included in the preoperative assessment of all morbidly obese patients at many medical facilities [7]. Hence, the importance of histopathological examination of the LSG specimen, particularly in the absence of routine OGD, remains uncertain [8].

Some studies suggest that LSG specimens should undergo gross examination only, with microscopic analysis reserved for cases with a history of significant disease or clinically relevant findings [9]. There is no clear consensus on whether preoperative endoscopy should be standard practice for LSG patients, nor whether histopathological examination of LSG specimens is necessary. This study aimed to evaluate the practice at our center and assess the histopathological findings in LSG specimens.

## Materials and methods

We performed a retrospective study to evaluate the histopathology results of all LSG procedures conducted at our center. Data were collected from January 1, 2017, to December 31, 2022, for patients undergoing LSG at Heartlands Hospital, University Hospitals Birmingham. To identify a suitable cohort, an electronic list of all operations performed by consultants during this period was generated. We searched for codes corresponding to laparoscopic sleeve gastrectomy and bariatric surgery. All patients aged 25-75 years who underwent LSG and had complete records available were included. The sampling technique was non-probability, consecutive sampling. Patients with missing records, those who underwent concomitant procedures, or those who had Roux-en-Y gastric bypass (RYGB) were excluded. Of the 310 cases retrieved, 107 were excluded (77 had RYGB, and 30 had incomplete records), leaving 203 cases for analysis. Operation notes were screened to ensure the patients met the inclusion criteria.

As this was a retrospective study without changes to patient care, it was registered as an audit on CARMS, and approval was obtained from the Ethical Review Committee (ERC). Data collection began after ERC exemption using a specifically designed proforma. A retrospective review of medical records and histopathology reports was conducted for all patients who met the selection criteria. Patient demographics and clinical presentations were reviewed from initial assessment forms, and intraoperative details were obtained from operative notes. Histopathology results were gathered from final pathology reports. Only the principal investigator and co-investigators had access to the data, with confidentiality maintained using coded identifiers. This study did not interfere with the patients' treatment, and the data collected were solely used for this analysis.

Descriptive statistics were used to summarize the baseline characteristics of the study population. Continuous variables, such as age and body mass index (BMI), were reported as mean and standard deviation (SD), while categorical variables, such as gender and preoperative OGD, were reported as numbers and percentages. Histopathological findings, including normal mucosa, chronic gastritis, *H. pylori*-associated gastritis, chronic gastritis with focal intestinal metaplasia, and gastrointestinal stromal tumor (GIST), were also reported as numbers and percentages. All data were analyzed using IBM SPSS Statistics for Windows, Version 27 (Released 2020; IBM Corp., Armonk, New York).

## Results

A total of 310 patients were screened, of which 203 were included in the study. The mean age of the patients was 45.7 years (±10.8). The majority of patients were female, accounting for 83% (n=168), while 17% (n=35) were male. The mean BMI of the patients was 45.4 (±7.3). Age distribution showed that 6% (n=14) were under 30 years of age, 25% (n=51) were between 31 and 40, 39% (n=79) were between 41 and 50, and 29% (n=59) were between 51 and 60. BMI distribution revealed that 4% (n=9) had a BMI under 35, 18% (n=36) had a BMI between 35 and 39.9, 38% (n=78) had a BMI between 40 and 44.9, 24% (n=49) had a BMI between 45 and 49.9, 10% (n=21) had a BMI between 50 and 54.9, and 5% (n=10) had a BMI between 55 and 59.9. Almost none of the patients underwent pre-operative OGD, with 99.5% (n=202) not receiving it. Only one patient had a pre-operative OGD, and the result was normal, as can be seen in the baseline characteristics data (Table 1).

**Table 1:** Baseline Characteristics BMI: body mass index; SD: standard deviation; OGD: oesophago-gastro-duodenoscopy.

Baseline characteristics	Value
Age (mean ± SD)	45.7 ± 10.8
BMI (mean ± SD)	45.4 ± 7.3
Age group, n (%)	
<30	14 (6)
31-40	51 (25)
41-50	79 (39)
51-60	59 (29)
BMI group, n (%)	
<35	9 (4)
35-39.9	36 (18)
40-44.9	78 (38)
45-49.9	49 (24)
50-54.9	21 (10)
55-59.9	10 (5)
Gender, n (%)	
Male	35 (17)
Female	168 (83)
Pre-op OGD, n (%)	
Yes	1 (0.5)
No	202 (99.5)

In the final histopathological reports, the majority of patients, 81.28% (n=165), had normal gastric mucosa. Chronic gastritis was observed in 14.29% (n=29) of cases. A very small number of patients, approximately 1% each, had more specific histopathological findings: 0.99% (n=2) had *H. pylori*-associated gastritis, 0.99% (n=2) had *H. pylori*-associated chronic gastritis, 0.99% (n=2) had chronic gastritis with focal intestinal metaplasia, and 0.99% (n=2) had GIST. Additionally, one patient (0.49%) was found to have a spindle cell lesion, as shown in Table 2.

**Table 2:** Histopathology Findings GIST: gastrointestinal stromal tumor; *H. Pylori*: *Helicobacter pylori.*

Histopathology findings	n (%)
Normal gastric mucosa	165 (81.28)
Chronic gastritis	29 (14.29)
*H. pylori-*associated gastritis	2 (0.99)
*H. pylori*-associated chronic gastritis	2 (0.99)
Chronic gastritis with focal intestinal metaplasia	2 (0.99)
GIST	2 (0.99)
Other	1 (0.49)

## Discussion

This study analyzed 203 patients out of an initial 310, focusing on those with complete records. The mean age was 45.7 years, with most patients being female (83%, n=168). The mean BMI was 45.4. Almost all participants (99.5%, n=202) did not undergo pre-operative OGD, with only one patient undergoing the procedure. The majority of patients (81.28%, n=165) had normal gastric mucosa, while approximately 14% (n=29) had chronic gastritis.

Bariatric surgery has proven to be a safe and effective treatment for obesity, leading to sustained weight loss, improvement or resolution of related health conditions, and significant increases in both life expectancy and quality of life. Given that the stomach is a primary focus of bariatric surgery, patients typically undergo thorough medical evaluations before surgery to minimize risks. However, there is ongoing debate regarding the necessity of routine upper gastrointestinal endoscopy (OGD) with biopsy in the preoperative evaluation, particularly for patients who do not present with significant gastrointestinal (GI) symptoms [10]. The European Association for Endoscopic Surgery (EAES) advocates for routine OGD before bariatric surgery, while the Society of American Gastrointestinal and Endoscopic Surgeons (SAGES) recommends considering OGD if there is suspicion of gastric issues prior to surgery [11].

In our study, pre-operative OGD was performed in only 0.5% (n=1) of patients, with normal results. In contrast, a similar study by Saafan et al. reported that nearly 100% (n=1555) of patients underwent pre-op OGD, and the majority had normal gastric mucosa or chronic gastritis [12]. In our study, 81% (n=165) of patients had normal gastric mucosa, while 14.29% (n=29) had chronic gastritis. Similarly, in a study by Giovanni et al., no significant pathological abnormalities were found in 82% of histopathology reports. Among these, 42% were normal, 25% showed proton pump inhibitor-related changes, and 15% had chronic gastritis [13].

In a study conducted by Husniye et al. with a sample size of 305 patients to assess the clinical significance of histopathology in LSG patients, it was found that 10.8% (n=33) had normal gastric mucosa, while 47.5% (n=145) had chronic inactive gastritis. Pre-operative endoscopy was performed in all these patients, and it returned normal results for 41.6% (n=127) [14]. Similarly, in another study by Sulaiman et al. analyzing histopathological specimens from 656 LSG patients, the results revealed that 74.4% (n=480) had chronic gastritis, 9.6% (n=63) showed follicular gastritis, and 1.8% (n=12) had atrophic gastritis [15]. A study by Ammiel et al. on histopathological findings in 925 LSG patients found that 99% of the histopathology results showed no significant findings beyond gastritis, and pre-operative endoscopy biopsy findings mirrored the LSG specimen analysis. This suggests that intra-operative examination of specimens might reduce costs and burden on the healthcare system [16].

A prospective study by Badr et al., involving 546 patients, found that fewer than 1% of patients had incidental benign lesions, with no malignancies detected. The study suggested that routine microscopic examination of LSG specimens may not be necessary, advocating instead for selective microscopic examination based on clinical history and macroscopic examination [17]. Another retrospective histopathological analysis of 613 LSG specimens by Stephaine et al. revealed that 52% had normal gastric mucosa, 33% had chronic gastritis, and 5% had Helicobacter-associated gastritis, with no malignancies detected [18].

There is still no consensus on whether pre-operative OGD alone is sufficient or whether LSG specimens should routinely be sent for histopathology. Some experts propose that if pre-op OGD is conducted, only a macroscopic examination of the LSG specimen is necessary, and microscopic examination should be reserved for cases with suspicious lesions. The aim of this study was to evaluate our practice and propose a pathway or plan for LSG candidates.

This was a single-center retrospective study with a smaller sample size, which may introduce an element of observer bias. Further prospective studies are required to establish a global consensus.

## Conclusions

Our five-year retrospective analysis suggests that routine histopathological examination of sleeve gastrectomy specimens usually reveals normal gastric mucosa or chronic gastritis, which are non-significant findings, suggesting that the routine submission of all LSG specimens for histopathological analysis may not be necessary in the majority of cases. Given the low yield of significant findings, the routine microscopic examination of all LSG specimens may represent an unnecessary burden on pathology services and healthcare resources. A more selective approach, guided by preoperative clinical evaluation, pre-op OGD, and post-operative gross examination of the specimen, could be a more efficient strategy. Further multicenter studies with larger cohorts are recommended to establish definitive guidelines and to better understand the role of histopathology in the management of patients undergoing LSG. This could ultimately lead to more tailored and cost-effective patient care in bariatric surgery.
